# Hyperglycemia in hospitalized patients of a tertiary care hospital: prevalence and treatment in two cross-sectional evaluations (2011-2020)

**DOI:** 10.20945/2359-3997000000452

**Published:** 2022-03-23

**Authors:** Patrícia Mendonça Oliveira Rosinha, Isabel Maria Ramos Inácio, Sofia Monteiro de Moura Teixeira, Cláudia Soares do Amaral, Maria Helena Cardoso Pereira da Silva

**Affiliations:** 1 Centro Hospitalar Baixo Vouga Departamento de Endocrinologia Aveiro Portugal Departamento de Endocrinologia, Centro Hospitalar Baixo Vouga, Aveiro, Portugal; 2 Centro Hospitalar e Universitário do Porto Departamento de Endocrinologia Porto Portugal Departamento de Endocrinologia, Centro Hospitalar e Universitário do Porto, Porto, Portugal

**Keywords:** Diabetes mellitus, glucose-lowering therapy, metabolic control, capillary blood glucose, in-hospital mortality

## Abstract

**Objective:**

A study at *Centro Hospitalar Universitário do Porto* in 2011 revealed suboptimal control of inpatient hyperglycemia and a similar one was carried out in 2020. This study compares the results of 2011 and 2020 regarding prevalence of hyperglycemia, metabolic control, treatment and glycemic profile by infection/non-infection diagnosis.

**Subjects and methods:**

We performed two cross-sectional studies on 13^th^ December 2011 and 9^th^ October 2020 that included all non-critical adults with at least 24 hours of hospitalization, with no specific intervention between them. Glycemic control evaluated by minimum and maximum capillary blood glucose (CBG) in the previous day categorized as hypoglycemia (<70 mg/dL), normoglycemia (70-179 mg/dL) and hyperglycemia (≥180 mg/dL) (SPSS v.20).

**Results:**

A total of 418 and 445 patients were respectively included in 2011 and 2020 studies and the prevalence of hyperglycemia was similar. Glycemic control improved numerically although not significantly in 2020: increase in normoglycemia, reduction in hyperglycemia and reduction in hypoglycemia. There was an increase in the use of basal-bolus regimens (19.6% vs. 7.3%, p = 0.009) and a decrease in human basal (p < 0.01) and rapid-acting insulin use (p = 0.001) with a proportional increase in long-acting (p = 0.002) and rapid-acting analogs (p < 0.001) use. There was a higher prevalence of infection (39.8% vs. 23.1%, p = 0.006) in 2020 and, in the infection subgroup, there were higher insulinization rates (37.3% vs. 10.7%, p = 0.017) and a trend to glycemic control improvement.

**Conclusion:**

Despite the higher insulinization rates, the preference for new insulin analogs and a trend to better glycemic control, we have not yet reached targets, so education still remains necessary.

## INTRODUCTION

Diabetes mellitus (DM) comprises a group of heterogeneous metabolic disorders whose main finding is chronically high values of blood glucose concentration ( 1 ). Hyperglycemia in hospitalized patients (with and without DM) is associated with adverse clinical outcomes in critically and non-critically ill patients, namely in terms of complications, longer length of stay (LOS) and mortality. Improvement of glycemic control translates into shorter LOS, lower rates of infection and risk of multiorgan failure as well as both short and long-term mortality ( 2 ). This knowledge has led to a growing worldwide concern with hyperglycemia and its repercussions ( 3 ).

Hyperglycemia in hospitalized patients is defined as blood glucose values above 140 mg/dL and current recommendations suggest that glucose levels persistently above this value might require evaluation and some intervention namely in terms of hyperglycemic agents. However, a glycated hemoglobin (HbA1c) ≥ 6.5% indicates that the onset of hyperglycemia preceded hospitalization and, in that case, a DM diagnosis should be considered. For the majority of hospitalized non-critical DM patients, the glycemic target to be achieved will be 140-180 mg/dL, although other ranges may be adequate in selected patients ( 4 ).

According to current guidelines, insulin remains the mainstay of treatment for in-hospital DM and the use of only a sliding scale insulin regimen is considered inappropriate in the hospital setting once it might be associated with poor glycemic control and thereafter higher infection rates, increased mortality and prolonged hospital stay. Therefore, the basal-bolus regimen is, according to these recommendations, the preferred treatment for non-critically ill hospitalized patients as long as they have good nutritional intake ( 4 – 10 ). Nonetheless, the best insulin regimen for hospitalized patients is still not consensual and there is recent evidence showing superiority of other regimens, namely only basal/basal-plus insulin, in terms of hyperglycemia control and without causing an increase in hypoglycemia. Surprisingly, these studies also show a greater benefit of using the sliding scale compared to the basal-bolus scheme due to the lower risk of hypoglycemia in some populations and the absence of significant differences regarding euglycemia/hyperglycemia ( 11 , 12 ).

In 2011, we detected a high prevalence of hyperglycemia among hospitalized patients in our institution and a low percentage of these patients were normoglycemic or under basal insulin regimen. ( 13 ). Since then, some training sessions were carried out to improve these aspects and the metabolic control of in-hospital diabetic patients, but the overall perception is that in-hospital hyperglycemia continues to be undervalued.

Therefore, it becomes important to understand the current reality of treating in-hospital hyperglycemia and to compare it with the previous evaluation in 2011.

The main aim of this study was to compare the results of two cross-sectional studies with similar methodology carried out at the same institution in different years (2011 and 2020) in terms of prevalence of hyperglycemia, metabolic control, treatment and glycemic profile by infection/non-infection diagnosis, looking for a change in the hyperglycemia treatment paradigm. As secondary outcomes, we also intended to investigate the association between variables related to glucose-lowering treatment (namely the type of insulin and type of insulin regimen) and others related to glycemic control and LOS.

## SUBJECTS AND METHODS

**Figure 1:**
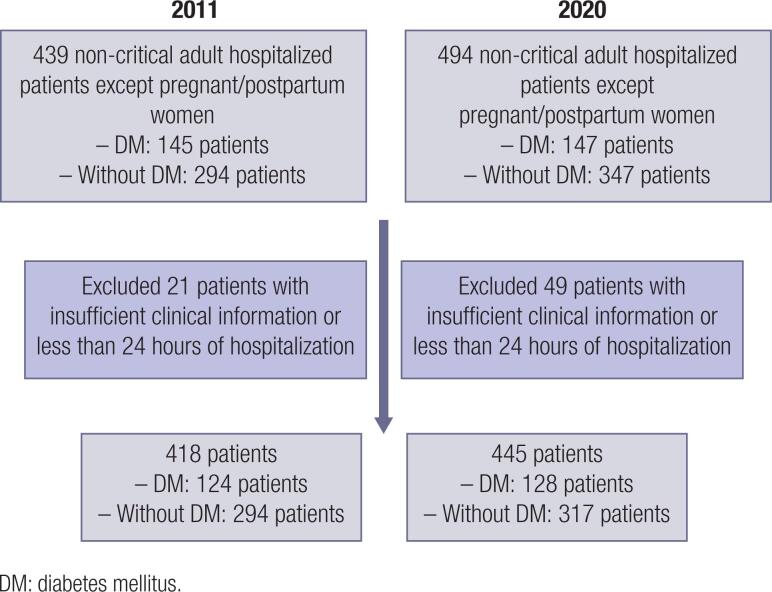
Study flowchart.

We performed two cross-sectional studies at *Centro Hospitalar Universitário do Porto* (CHUP): the first was carried out on 13^th^ December 2011, the second on 9^th^ October 2020 and no specific intervention was made between them except for the training sessions usually provided in a similar proportion. CHUP is a public sector university tertiary care hospital in Portugal with an inpatient capacity of 550 patients in 2011 and 791 in 2020. We included all non-critical adult patients admitted to our institution with a minimum of 24 hours of hospitalization. Pregnant/postpartum women and patients with insufficient clinical information in the process were excluded ( Figure 1 ). The study protocol was approved by the Hospital's Ethics Committee.

Data was collected anonymously from the electronic clinical record or directly with the patient (in case of missing information) concerning demographic information, LOS, and DM diagnosis.

The diagnosis of in-hospital hyperglycemia/DM was confirmed by consulting the patient's clinical file. From patients with hyperglycemia, we also collected information regarding type of DM, infection/non-infection diagnosis, main diagnosis (related/non-related to DM), capillary blood glucose (CBG) monitoring the day before, minimum and maximum CBG in the previous 24 hours and in-hospital glucose-lowering therapy. The diagnosis of infection was considered if the patient was under antibiotic treatment (except for prophylaxis) or presented clinical or analytical signs of infection (different criteria were considered according to the type of infection). Glycemic control was evaluated by the average of the minimum and maximum CBG in the previous day, categorized in: hypoglycemia (<70 mg/dL), normoglycemia (70-179 mg/dL) and hyperglycemia (≥180 mg/dL). The institution does not have a uniform protocol for assessing the glycemic control of hospitalized patients, so this evaluation is carried out by the Assistant Medical Team and, if necessary, the support of the Endocrinology Department may be requested in an internal consultation regimen. For the secondary outcome analysis, a categorization of the LOS variable into < 15 days and ≥ 15 days was used.

The glycemic profile and glucose-lowering treatment were also compared in the infection/non-infection subgroups in each of the years.

Data analysis was performed using the statistical package IBM SPSS Statistics, version 20.0.0 (IBM Corp., Armonk, NY, USA). Categorical variables are presented as frequencies and percentages and continuous variables as means and standard deviations (SD) or medians and interquartile ranges (IQR) for variables with skewed distributions. Normal distribution was checked using Shapiro-Wilk test or skewness and kurtosis. All reported p-values are two-tailed, with a p < 0.05 indicating statistical significance. Categorical variables were compared with the Pearson's Qui-square test or Fisher's exact test as appropriate. For the analysis of secondary outcomes, a qui-square odds ratio (OR) was calculated for each of the found associations. Continuous variables were compared with independent samples t-test or Mann-Whitney U-test (if skewed distribution).

## RESULTS

A total of 418 and 445 patients were respectively included in 2011 and 2020 studies, of which 294 and 317 did not have DM diagnosis ( Figure 1 ). There was a non-significant reduction in the number of hospitalized patients with hyperglycemia in 2020 compared to 2011 (28.8% vs. 29.7%, p = 0.822). The vast majority of diabetic patients included in both cross-sectional studies had type 2 DM (94.5% in 2020 and 93.5% in 2011) although there were also patients with the following: type 1 DM, DM induced by glucocorticoids (GC), DM secondary to disease of the exocrine pancreas and a minority not yet classified ( Table 1 ). There was a preponderance of male patients in both studies (57.8% in 2020 and 56.5% in 2011, p = 0.899). Patients included in the 2020 study were, on average, older than those of 2011 at time of data collection (p = 0.069). There were no significant differences regarding the LOS between groups. In what concerns diagnosis, there was a significantly higher prevalence of infection (39.8% vs. 23.1%) and a non-significant lower proportion of diagnoses related to DM (5.5% vs. 9.1%) in 2020 ( Table 1 ).

**Table 1:** Demographic description of patients with in-hospital hyperglycemia by year of study

		2011	2020	p value
In-hospital hyperglycemia – n (%)		124 (29.7)	128 (28.8)	0.822 *
DM classification – n (%)				
	Type 1	2 (1.6)	4 (3.1)	0.734 *
	Type 2	116 (93.5)	121 (94.5)	
	Induced by GC	3 (2.4)	2 (1.6)	
	Not yet clarified	2 (1.6)	1 (0.8)	
	Diseases of the exocrine pancreas	1 (0.8)	0	
Gender – n (%)				
	Male	70 (56.5)	74 (57.8)	0.899 *
	Female	54 (43.5)	54 (42.2)	
Age at time of data collection (years) – mean ± SD		71.0 ± 11.9	73.7 ± 11.6	0.069 a
	minimum – maximum	34 - 92	32 - 95	
LOS – n (%)				
	A) 1-3 days	21 (17.4)	22 (17.2)	0.287 b
	B) 4-7 days	33 (27.3)	22 (17.2)	
	C) 8-14 days	26 (21.5)	32 (25.0)	
	D) 15-30 days	28 (23.1)	30 (23.4)	
	E) >30 days	13 (10.7)	22 (17.2)	
Infection diagnosis – n (%)				
	Yes	28 (23.1)	51 (39.8)	0.006 *
	No	93 (76.9)	77 (60.2)	
Type of infection diagnosis – n (%)				
	COVID-19	0	3 (5.9)	0.505 b
	Other respiratory infections	10 (35.7)	9 (17.6)	
	Urinary	5 (17.8)	7 (13.7)	
	Abdominal	1 (3.6)	4 (7.8)	
	Cutaneous	2 (7.1)	7 (13.7)	
	Pelvic	1 (3.6)	1 (2.0)	
	Sepsis	6 (21.4)	3 (5.9)	
	Osteoarticular	1 (3.6)	4 (7.8)	
	Cardiac	1 (3.6)	0	
	Cerebral	0	2 (4.0)	
	Undetermined	1 (3.6)	11 (21.6)	
Main diagnosis – n (%)				
	Related to DM	11 (9.1)	7 (5.5)	0.331 *
	Non-related to DM	110 (90.9)	121 (94.5)	

COVID-19: coronavirus disease; DM: diabetes mellitus; GC: glucocorticoids; LOS: length of stay; SD: standard deviation.

* Pearson's Qui-square test.

a Independent samples t-test.

b Fisher's exact test.

Table 2 compares glycemic control of patients with in-hospital hyperglycemia by year of study. In 2020, CBG monitoring was performed in a lower proportion of patients (85.0% vs. 93.5%, p = 0.062) and there was a non-significant improvement in glycemic control, with a numeric reduction in maximum CBG value (226.9 ± 77.2 mg/dL vs. 239.6 ± 68.4 mg/dL), an increase in the proportion of patients in normoglycemia (59.1% vs. 52.2%) and a reduction in the percentage in hyperglycemia (40.9% vs. 47.8%). Moreover, there were non-significant reductions in the percentage of patients having at least one hypoglycemia (1.8% vs. 6.9%) and one hyperglycemia (70.0% vs. 75.9%) ( Table 2 ).

**Table 2:** Glycemic control of patients with in-hospital hyperglycemia by year of study

		2011	2020	p value
CBG monitoring – n (%)		116 (93.5)	110 (85.0)	0.062 *
Minimum CBG value (mg/dL) – median (IQR)		119.5 (64.0)	124.0 (45.0)	0.133 c
Maximum CBG value (mg/dL) – mean ± SD		239.6 ± 68.4	226.9 ± 77.2	0.229 a
Average of minimum and maximum CBG values (mg/dL) – median (IQR)		176.5 (68.4)	168.5 (74.8)	0.608 c
Average of minimum and maximum CBG values by categories – n (%)				
	Normoglycemia (70-179 mg/dL)	60 (52.2)	65 (59.1)	0.348 *
	Hyperglycemia (≥180 mg/dL)	55 (47.8)	45 (40.9)	
At least 1 CBG value <70 mg/dL – n (%)		8 (6.9)	2 (1.8)	0.103 b
At least 1 CBG value ≥180 mg/dL – n (%)		88 (75.9)	77 (70.0)	0.369 *
At least 1 CBG value ≥300 mg/dL – n (%)		29 (25.0)	24 (21.8)	0.639 *

CBG: capillary blood glucose; IQR: interquartile range; SD: standard deviation.

* Pearson's Qui-square test.

a Independent samples t-test.

b Fisher's exact test.

c Mann-Whitney U-test.

Aspects related to the in-hospital treatment of hyperglycemia are shown in Table 3 . Despite the trend towards a lower proportion of patients on glucose-lowering therapy in 2020 (89.1% vs. 90.3%, p = 0.837), there was an increase in the use of basal-bolus regimens (19.6% vs. 7.3%, p = 0.009). The sliding scale was, as in 2011, the most prescribed type of regimen, despite the numeric reduction in its use (57.0% vs. 66.4%, p = 0.165). Moreover, the percentage of human basal and rapid-acting insulin use decreased in 2020 (respectively 8.7% vs. 50.0%, p < 0.001 and 75.7% vs. 92.8%, p = 0.001) with a proportional increase in the use of long-acting (91.3% vs. 50.0%, p = 0.002) and rapid-acting analogs (24.3% vs. 5.4%, p < 0.001). The number of patients exclusively on glucose-lowering drugs in hospital increased in 2020 (5.5% vs. 2.4%, p = 0.334) ( Table 3 ).

**Table 3:** Description of in-hospital hyperglycemia treatment by year of study

		2011	2020	p value
Under glucose-lowering therapy – n (%)		112 (90.3)	114 (89.1)	0.837 *
Anti-hyperglycemic agents – n (%)				
	None	12 (9.7)	14 (10.9)	0.837 *
	Only antidiabetic agents	3 (2.4)	7 (5.5)	0.334 b
	Antidiabetic agents + sliding scale	20 (16.1)	8 (6.3)	0.016 *
	Only sliding scale	52 (41.9)	53 (41.4)	1.000 *
	Basal insulin ± rapid-acting insulin	30 (24.3)	39 (30.4)	0.399 *
	Antidiabetic agents + basal insulin ± rapid-acting insulin	4 (3.2)	7 (5.5)	0.539 *
	Intravenous insulin infusion	3 (2.4)	0	0.247 b
Insulin regimen – n (%)				
	Sliding scale	73 (66.4)	61 (57.0)	0.165 *
	Basal insulin + sliding scale	26 (23.6)	25 (23.4)	1.000 *
	Basal-bolus	8 (7.3)	21 (19.6)	0.009 *
	Intravenous insulin infusion	3 (2.7)	0	0.240 b
Type of glucose-lowering drugs – n (%)				
	Metformin	20 (16.1)	10 (7.8)	0.050 *
	Sulfonylureas	8 (16.1)	1 (0.8)	0.017 b
	DPP4 inhibitors	0	10 (7.8)	0.002 b
	GLP1 agonists	0	3 (2.3)	0.248 b
	SGLT2 inhibitors	0	6 (4.7)	0.030 b
	Other classes	6 (4.8)	0	0.012 b
Type of basal insulin – n (%)				
	NPH	17 (50.0)	4 (8.7)	<0.001 *
	Long-acting analogs	17 (50.0)	42 (91.3)	0.002 *
Type of rapid-acting insulin – n (%)				
	Regular	103 (92.8)	81 (75.7)	0.001 *
	Rapid-Acting Analogs	6 (5.4)	26 (24.3)	<0.001 *

DPP4: dipeptidyl peptidase-4; GLP1: glucagon-like peptide-1; NPH: neutral protamine Hagedorn; SGLT2: sodium glucose cotransporter 2.

* Pearson's Qui-square test.

b Fisher's exact test.

Regarding the analysis by infection/non-infection diagnosis, there were no significant differences in the minimum and maximum values for CBG between those subgroups of both years. The rates of anti-hyperglycemic agents use was similar in 2011 and 2020 in both infection and non-infection subgroups (respectively, 89.3% vs. 88.2%, p = 1.000; 90.3% vs. 89.6%, p = 1.000). The subgroup diagnosed with infection in the 2020 sample showed non-significantly lower rates of antidiabetic agents use (13.7% vs. 28.6%, p = 0.137) and exclusive use of sliding scale (37.3% vs. 46.4%, p = 0.478) and significantly higher rates of insulinization (37.3% vs. 10.7%, p = 0.017). Concomitantly, there was also an increase in the proportion of patients in normoglycemia in the infection subgroup (67.2% vs. 22.0%, p = 0.166) in spite of the increase in the percentage in hyperglycemia (47.8% vs. 26.0%, p = 0.007) in 2020. Instead, in the group without infection, the opposite was found: a decrease in the proportion of patients in normoglycemia (32.8% vs. 78.0%, p = 0.166) and hyperglycemia (52.2% vs. 74.0%, p = 0.007) in 2020.

In the secondary outcome analysis, we found associations between the use of a sliding scale regimen and both an average maximum and minimum glucose value in the normoglycemia range compared to other insulin regimens (OR 2.649, p = 0.001) and a LOS of less than 15 days (OR 2.829, p < 0.001).

## DISCUSSION

The in-hospital prevalence of hyperglycemia did not vary significantly in the two time points studied contrary to what was expected considering the increase in the prevalence of diabetes in Portugal and worldwide and also the observed increase in the mean age of hospitalized patients in 2020 consistent with the increase in life expectancy that we have been witnessing globally ( 14 – 16 ). Data from the 2019 National Diabetes Observatory Annual Report showed a 39.3% increase in the number of hospitalized patients diagnosed with DM between 2009 and 2018 and, when analyzing international data, we found results of in-hospital prevalence of DM comparable to those of the present study ( 17 , 18 ).

Globally, there was a trend to better glycemic control in 2020: more patients in the normoglycemia range and fewer patients on the hyperglycemia range. This improvement reflects the increasing awareness of health care professionals about glycemic control in hospitalized patients, for which the training sessions provided during these last nine years have contributed. It is also important to consider the support provided by the Endocrinology Department in 2011 and 2020, which was reflected in an increase in the annual number of internal consultations from 955 to 3031 and its subsequent impact on glycemic control. In addition, the increased use of basal-bolus regimens and the preference for newer insulins with more physiologic profiles and lower risk of hypoglycemia may also have contributed to these results, namely for the reduction in episodes of hypoglycemia observed during hospitalization.

Current recommendations and evidence from previous randomized controlled trials are in favor of considering insulin as the most effective agent in controlling blood glucose at the hospital level ( 7 ). Nonetheless, it is interesting to note that non-insulin glucose-lowering drugs, not only DPP4 inhibitors and SGLT2 inhibitors but also GLP1 analogues, are starting to be used in hospital diabetes management. The use of antidiabetic agents is common in clinical practice and, with the emergence of new pharmacological classes, this therapeutic option has become even more attractive. Non-insulin drugs provide a decrease in the fluctuation of glucose levels, important non-glycemic benefits, while also being associated with a lower risk of hypoglycemia compared to insulin therapy. On the other hand, as potential limitations to its use, it is worth mentioning the delay and unpredictability of onset of action. Overall, by weighing the risks and benefits inherent to its use, some studies refer to the fact that this could be the time for a change in the paradigm of treating in-hospital hyperglycemia and that antidiabetic agents can be used to achieve appropriate glycemic control in some populations ( 2 , 19 , 20 ).

Despite the trend observed, we still found that almost forty percent of these patients are hyperglycemic during hospitalization. This can be due to the lack of treatment observed in almost ten percent of patients or, in light of current recommendations ( 21 ), mainly to the persistent high use of sliding scale regimen. By comparing our results with those of other hospitals in Portugal, we realized that the percentages of sliding scale schemes and glucose-lowering drugs use at hospital are similar or even higher ( 22 ). On the other hand, considering the results obtained by Sadhu and cols. ( 11 , 12 ), the most likely explanation for these results would be the reduced rates of use of only basal/basal-plus insulin.

This comparative study also shows a higher prevalence of diagnosis of infection in 2020, which can be framed in the context of the COVID-19 (coronavirus disease) pandemic. Notably, it was in the infection group that we observed a significant increase in the use of basal insulin regimens even though glycemic control has not improved in proportion in this group. This is a positive aspect albeit it reflects the need to further optimize therapeutic insulin regimens. In a global way, it seems that we managed to approach the recommendations even though not completely.

Our results also show an association between the use of a sliding scale regimen and an average maximum and minimum glucose value in the normoglycemia range compared to other insulin regimens, which is in accordance with the point of view of Migdal and cols. and Sadhu and cols. ( 11 , 12 ) even though contradictory to current recommendations. Moreover, we also found an association between the use of sliding scale insulin and a lower LOS, meaning that patients that achieved normoglycemia with only sliding scale regimen more often had a LOS inferior to 15 days. These results are in agreement with existing evidence that sliding scale insulin might be appropriate to use at the hospital level as long as for short periods of time ( 23 ) and this point may constitute a middle ground between current recommendations and recent evidence.

As limitations of this study, we must also mention the fact that only the minimum and maximum glucose values in the evaluated 24-hour period were available from the 2011 patient sample, which made it impossible to use the average of all the glucose values as the main glycemic control indicator. In addition, it compares two studies of cross-sectional nature, which only allows two punctual assessments of prevalence, glycemic control and treatment paradigm. Nonetheless, this particularity also gives originality to this study by allowing the comparison of the analysed parameters at two different time points, which is rarely found in previous studies evaluating the prevalence of in-hospital hyperglycemia. It is also important to mention that one of the time points evaluated overlapped the COVID-19 pandemic period in Portugal, which allowed the assessment of glycemic control at a time when there was an increase in the prevalence of infection in hospitalized patients, being this beneficial for the analysis in the infection/non-infection subgroups. Finally, this study focuses on an important and still controversial issue and shows real-life data from a tertiary hospital in Portugal.

In conclusion, these results might reflect a growing concern with this issue at the institutional level, as happened worldwide in recent years ( 2 ). Despite the improvement of several aspects, we have not yet reached satisfactory glycemic control of in-hospital hyperglycemia and much remains to be done, namely with regard to the use of the sliding scale insulin regimen particularly if long-term, in which case it stands out for low efficiency and potential harms ( 24 , 25 ). As an alternative to sliding scale, it would be a better option to prescribe a basal insulin or basal-plus/basal-bolus schemes according to current recommendations ( 4 ), or eventually consider the use of non-insulin glucose-lowering drugs in patients with no contraindications, an option that has been acquiring increasing importance at the hospital level, although its routine use is not yet recommended. These results stress the need to: a) increase the awareness of glycemic control in hospitalized patients; b) define protocols to manage in-hospital diabetes; c) provide education in these issues; and d) do periodic audits.
